# Massive ear keloids: Natural history, evaluation of risk factors and recommendation for preventive measures – A retrospective case series

**DOI:** 10.12688/f1000research.9504.2

**Published:** 2017-03-21

**Authors:** Michael Tirgan

**Affiliations:** 1 Keloid Research Foundation, New York, NY, 10023, USA

**Keywords:** Ear Keloid, Cryotherapy

## Abstract

Keloid disorder (KD) is an inherited wound healing ailment, frequently seen among Africans /African Americans and Asians.  Genetics of this disorder continues to be obscure and poorly understood.  Clinical manifestation of KD is quite variable and very diverse, spanning from individuals with one or very few small keloidal lesions, to those with numerous and very large lesions covering large portion of their skin. Ears are common locations for development of keloids.  Ear piercing is by far the leading triggering factor for ear keloid formation in genetically predisposed individuals. Although there are numerous publications about ear and earlobe keloids, there is a void in medical literature about massive ear keloids.  This paper focuses on the natural history of massive ear keloids and risk factors that lead to formation of these life-changing and debilitating tumors and recommendations for prevention.

## Introduction

Patients with keloid disorder (KD) carry a genetic abnormality that predisposes them to the disorder ^1^. Although no convincing genetic abnormalities have been linked to KD, clinical observation suggests that the genetic predisposition to KD has a broad spectrum ^2^. Individuals who suffer from mild form of the disorder typically develop one or few slow-growing keloidal lesions, whereas individuals with the severe form of the disorder often develop several large keloids. Perhaps the strongest link to the genetic underpinning of KD is observed in patients with Rubinstein-Taybi Syndrome, whereby a significant percentage of these patients develop keloidal skin lesions ^3^.

**Figure 1.**
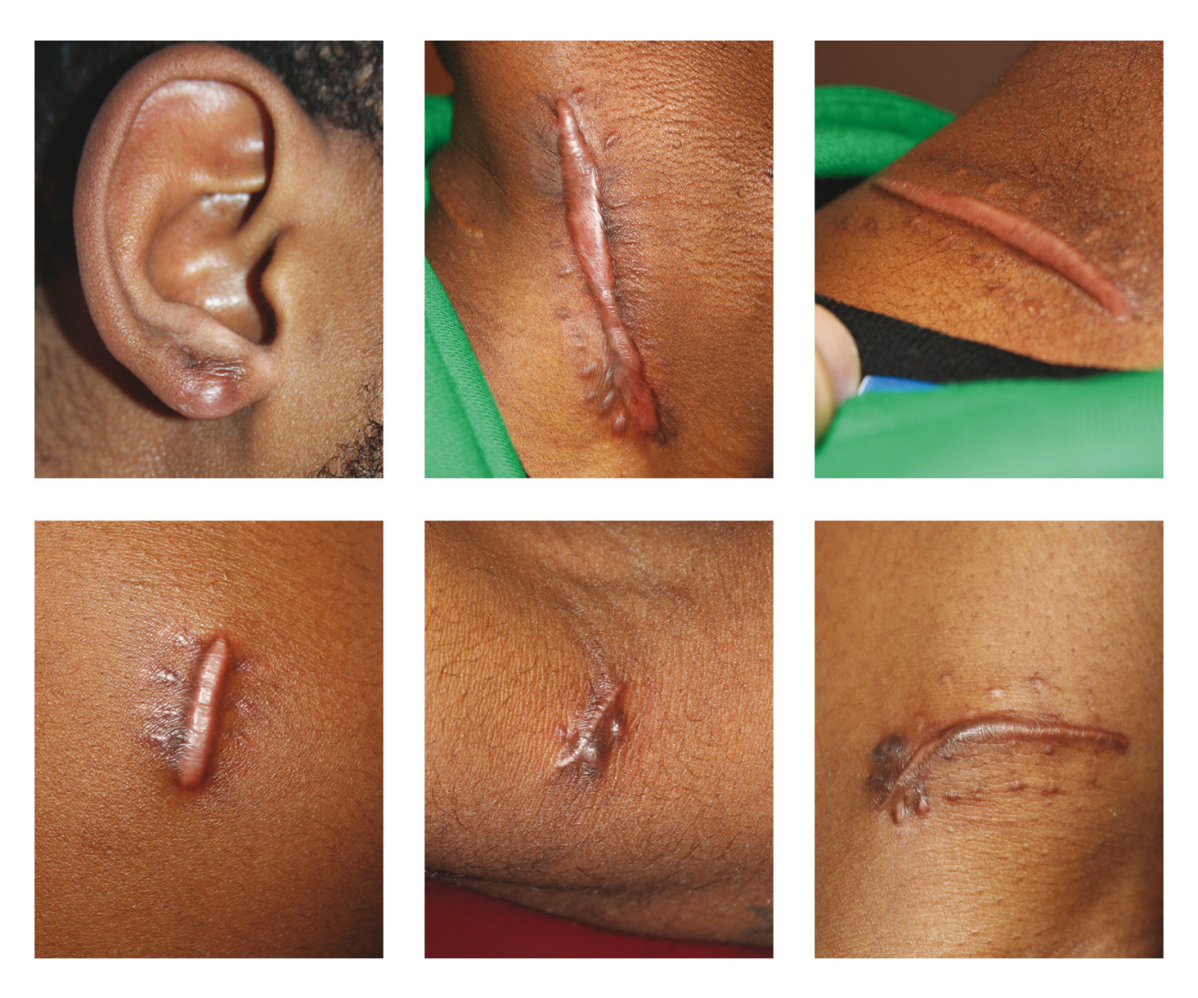
Young African American male with several keloidal skin lesions on various parts of his skin. Notice that each wounded area of skin has transformed into a keloidal lesion.

In addition to the genetics, other factors also play important roles in clinical presentation of KD. Most importantly, there must exist an injury to the skin that would trigger abnormal wound healing response that leads to the formation of keloid lesions ^2^. Figure 1 depicts a young African American male who developed an earlobe keloid following the piercing of his right ear. In addition, he also sustained several sharp and deep injuries to his neck, left shoulder and left arm. All wounded areas subsequently transformed into linear keloids. Therefore, it is safe to conclude that had he not pierced his ear or sustained other injuries, he would not have developed any of these keloids and would have remained completely asymptomatic. Therefore, simple clinical observations of this one patient teaches us that certain individuals harbor the KD genetic abnormality yet remain asymptomatic only because they have not pierced their ears or sustained a serious injury to their skin.

Another important fact about KD, which is well exemplified in this case, is that adjacent and even distant skin are also affected by the keloidal process; thus, the wounding of normal-appearing skin will inevitably lead to the formation of new keloid lesions.

In addition to genetics and skin injuries, the third important factor in the clinical presentation of KD is the age of the individual. The peak age of onset of KD occurs during puberty; however, certain types of skin injuries only occur later in life. For instance, the typical age of those undergoing cardiac bypass surgery or facelift surgery is in 6th and 7th decade of life. As such, certain KD carriers will remain asymptomatic until they undergo their first surgery and end up with chest-wall or peri-auricular keloids ^1, 4^. Race, gender, passage of time and therapeutic interventions are other important factors that play their own unique roles in clinical presentation of this disorder. The wide spectrum of these factors contributes to highly variable phenotype of KD. The clinical presentation of KD is to some extent race and gender dependent. Large and tumoral keloids, including massive ear lesions, are more often encountered among Africans, African Americans and individuals with black skin ^2^.

Focusing our attention to the ears, it is common knowledge that keloid lesions grow over time. With medical interventions, some KD lesions respond well to the treatments, but some lesions fail to respond, or even get worse and grow much larger. By far, the most important factor in development of all primary keloidal lesions is the initial wounding injury of the skin. However, the surgical removal of ear keloids that is commonly performed by ENT specialists, plastic surgeons and general dermatologists, defies this very basic principal of keloid formation. The extent of the injury to the surrounding skin when an ear keloid is surgically removed is obviously several fold greater than the primary injury sustained from the piercing procedure. This iatrogenic injury will undoubtedly trigger a keloidal wound healing response that is not only more intense than the one triggered by the original piercing event but also much greater in magnitude and distribution. Studies have indicated that almost all ear keloids and almost all other keloid lesions will relapse after surgery; hence, the need for adjuvant treatment has been emphasized by almost every author who has published on this topic. Adjuvant treatments in the form of post-operative steroid injections ^5^, pressure devices ^6^ or even radiation therapy ^7, 8^ are often incorporated in management of ear keloids in order to counteract the fully expected keloid recurrence after surgery. However, despite the meticulous use of all available adjuvant treatments, a large number of patients will suffer from recurrent ear keloids and undergo second, third or fourth surgeries. Unfortunately, the ear keloids will continue to relapse in many instances. At some point, the surgeon, the patient, or even both will abandon therapeutic interventions.

This article focuses on these unfortunate cases; instances of recurrent large, semi-massive, and massive ear keloids among mostly young patients who ultimately accept the reality that surgery and/or adjuvant radiation therapy cannot treat their keloids, thereby resigning themselves to living with huge tumoral keloids hanging from their ears, an unwanted and unpleasant outcome that impacts every aspect of their daily lives.

## Materials and methods

This is a retrospective analysis of 283 consecutive patients with ear keloids who were seen by the author in his keloid specialty medical practice. Patients with post-otoplasty ear keloids (n=14), and those with post-facelift peri-auricular keloids were not included in this study as the triggering factor for these keloids, i.e. primary surgery, clearly results in a much larger injury to the ear tissue as compared to the injury from ear piercing. Author intends to publish and report these patients in a separate manuscript.

Data was analyzed using descriptive statistics. Number of patients and percentages were computed for each category. To test the differences in each dataset category, general z-test were computed. The 95% confidence intervals for the observed proportions were calculated by Clopper-Pearson method. Statistical analysis was performed using MedCalc 15.8.

The underlying research project for this retrospective study was determined by the Western IRB to meet the conditions for exemption under 45 CFR 46.101(b)(4). Consent is not required for studies that are determined to be exempt under 45 CFR 46.101(b)(4).

## Results

Keloids were assessed visually and categorized according to their size into four separate groups.

**Figure 2.**
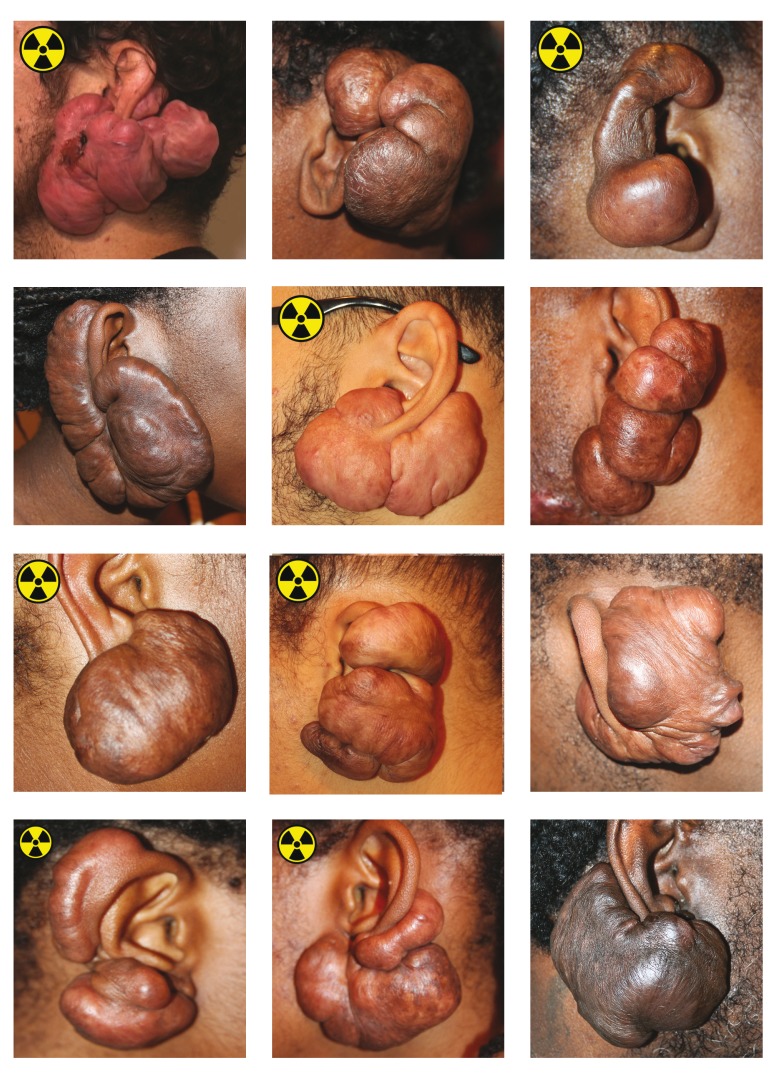
Massive ear keloids are larger than the size of corresponding ear. Yellow radiation signs identify patients who have previously received adjuvant radiation therapy after removal of their ear keloids.

**Figure 3.**
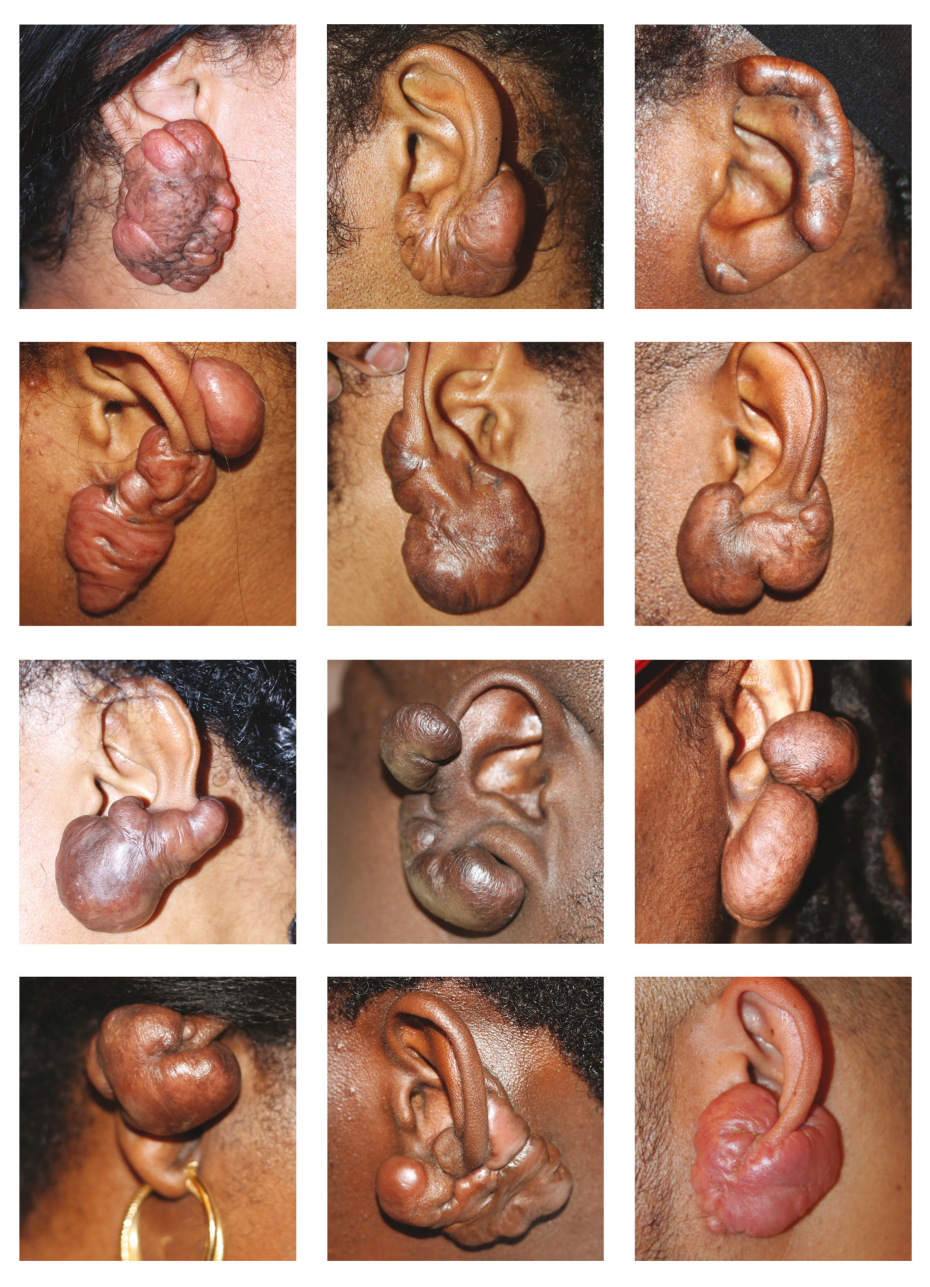
Semi-massive ear keloids measure at least half the size of the corresponding ear.

**Figure 4.**
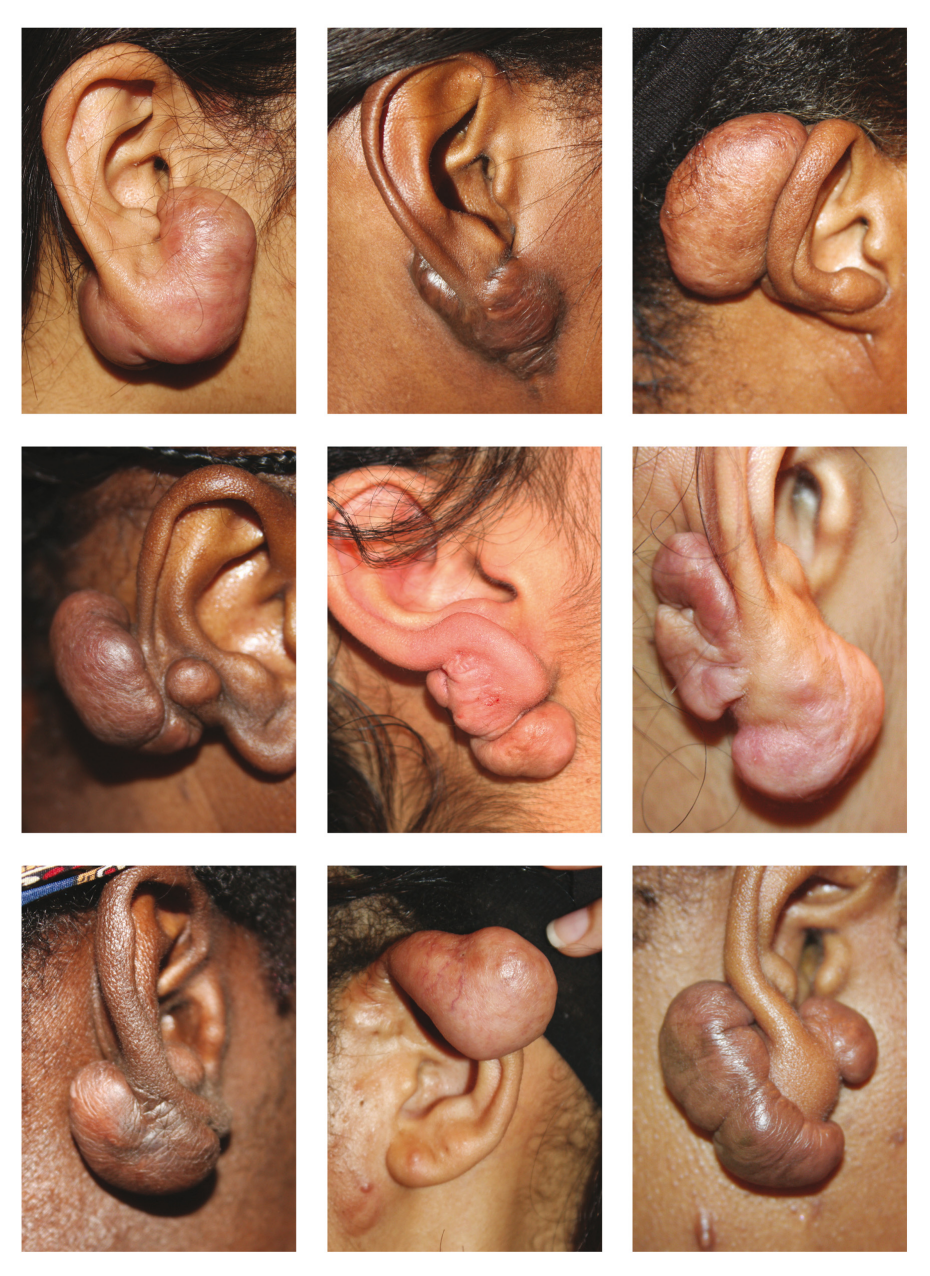
Large ear keloids measure larger than the corresponding earlobe.

**Figure 5.**
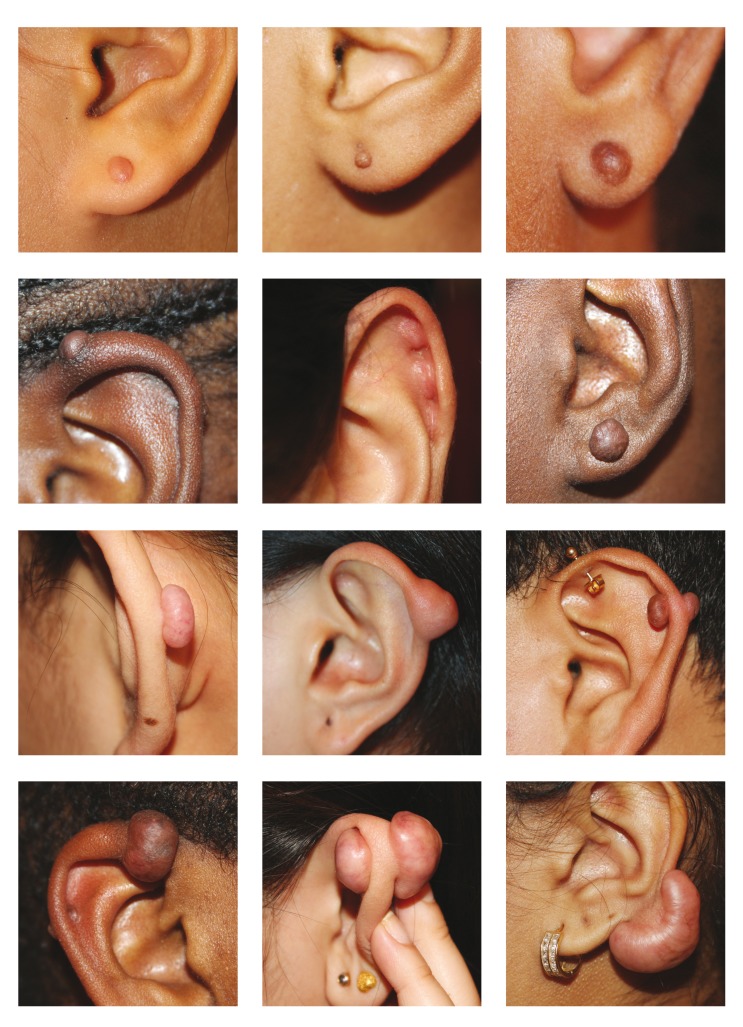
Early stage, primary ear keloids in various stages of development.

**1- **  **Massive ear keloids**: the size of the keloid mass is greater than the surface area of the corresponding ear. Thirteen patients (4.6%) met this criterion. Three patient were Caucasians, and 10 were African Americans. Four patients (three females and one male) had bilateral massive ear keloids. Figure 2 depicts several patients in this category.
**2- **  **Semi-massive ear keloids**: the size of the keloid mass is at least 50% of the surface area of the corresponding ear, but smaller than massive ear keloids. Eighteen patients (6.4%) met this criterion. Two patients were Caucasians, and sixteen were African Americans. Figure 3 depicts several patients in this category.
**3- **  **Large ear keloids**: the size of the keloid mass was more than the size of the corresponding earlobe, but smaller than semi-massive ear keloids. In total, 181 patients (64%) met this criterion. Forty-nine patients were Caucasians or Asians, and 132 patients were African Americans. Figure 4 depicts several patients in this category.
**4- **  **Small ear keloids**: the size of the keloid mass is less than the size of the corresponding earlobe. Seventy-one patients (25%) met this criterion. Twenty-eight patients were Caucasians or Asians, and 43 patients were African Americans. Figure 5 depicts several patients in this category.

Table 1 summarizes characteristics of the patients within each group.

**Table 1.** Patient characteristics.

Patients N=	283
**Asians/Caucasians**	**82** (29%)
Male	30
Female	52
**African Americans**	**201** (71%)
Male	79
Female	122
**Massive ear keloids**	**13 (4.6%)**
Gender Male	6
Female	7
Race Caucasian - Asian	3
African American	10
**Semi-massive ear** **keloids**	**18 (6.4%)**
Gender Male	6
Female	12
Race Caucasian - Asian	2
African American	16
**Large ear keloids**	**181** (64%)
Gender Male	72
Female	109
Race Caucasian - Asian	49
African American	132
**Small ear keloids**	**71 (25%)**
Gender Male	25
Female	46
Race Caucasian - Asian	28
African American	43

Other than author’s recently published keloid staging system ^8^, there are no other previously described methodologies that would allow for more precise grouping of the ear keloids. Table 2 shows the stage classification of solitary ear keloids according to the this staging system.

**Figure 6.**
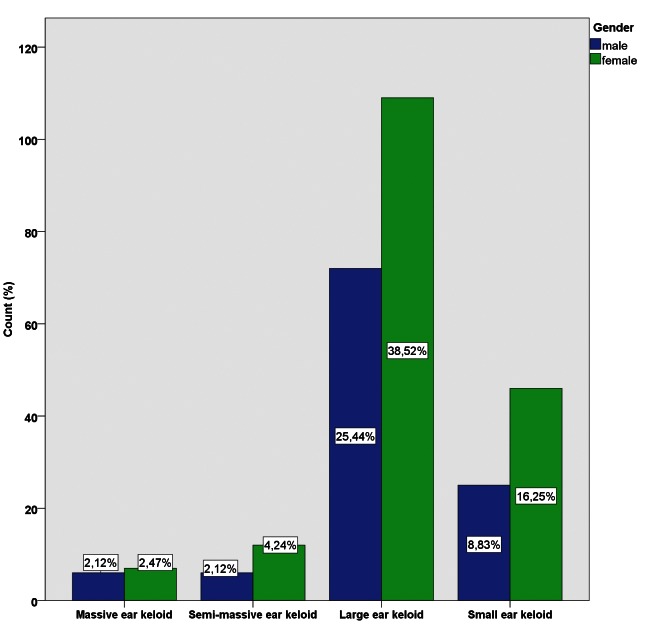
Proportion of keloid patients in each study group according to gender.

Proportionally, more females were noted among each study group ( Figure 6), however, this finding may simply be related to the fact that more women pierce their ears. The proportional difference between females and males is statistically significant and shown in Table 3 (95% CI of observed proportion for female gender is 55.05%-66.72%, Z=3.701, P=0.0002).

**Figure 7.**
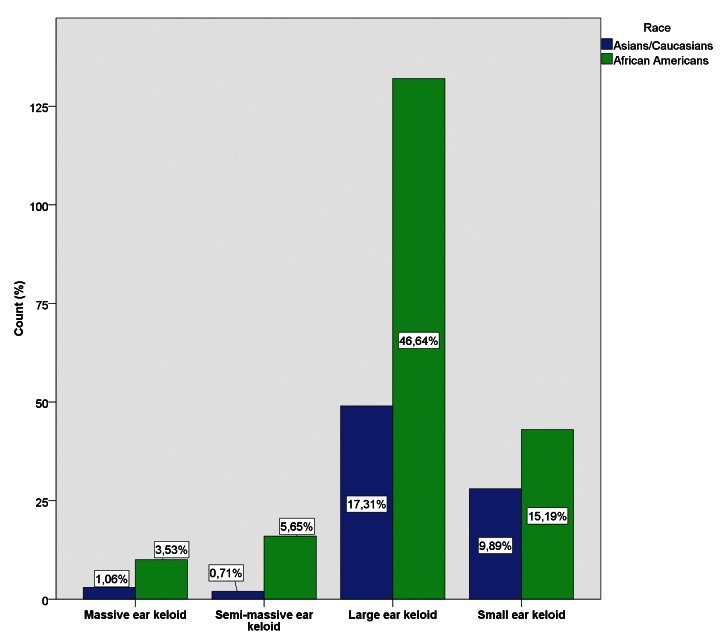
Proportion of keloid patients in each study group according to race.

Proportionally, there were more Africans/African Americans among each study group ( Figure 7).

**Table 2.** Stage grouping for patients with solitary ear keloids.

Massive ear keloid	Stage 1C	Presence of only one keloidal lesion that measures greater than 10 centimeters in any dimension.
Large and Semi-massive ear keloids	Stage 1B	Presence of only one keloidal lesion that measures 2.1 – 10 centimeters in any dimension.
Small ear keloids	Stage 1A	Presence of only one keloidal lesion that measures no greater than 2 centimeters in any dimension.

Furthermore, African/African American race was noted to be a major risk factor for development of massive and semi-massive ear keloids. The proportional difference between the two racial groups is statistically significant and shown in Table 4 (95% CI of observed proportion for African American race is 66.34%-94.62%, Z=3.786, P=0.0002).

**Table 3.** Proportion of females v. males among all study subjects.

Gender	N	Observed proportion	95% CI of observed proportion	Test proportion	Z-statistics	P (2-sided)
Female	174	61%	55.05%–66.72%	0.5	3.701	0.0002
Male	109	39%				
Total	283	100%				

Prior keloid removal surgery was the most important risk factor among all patients with massive and semi-massive ear keloids. Without exception, all these patients had undergone anywhere between one to seven prior keloid removal surgeries. Prior keloid removal surgery was also the most important risk factor among patients with large ear keloids. Table 5 shows the proportion of patients having undergone ear keloid removal surgery in each of these three study groups.

**Table 4.** Proportion of patients with massive and semi-massive ear keloids according to their race.

Race	N	Observed proportion	95% CI of observed proportion	Test proportion	Z-statistics	P (2-sided)
African American	26	84%	66.34%–94.62%	0.5	3.786	0.0002
Caucasian-Asian	5	16%				
Total	31	100%				

**Table 5.** History of prior ear keloid removal surgery.

Massive Ear Keloids						
	N	Proportions	95% CI	Test proportion	Z-statistics	P (2-Sided)
Surgery	13	100%	75.29%–100.00%	0.5	3.606	0.0003
No Surgery	0	0.00%				
Total	13	100%				
**Semi-Massive Ear Keloids**						
	N	Proportions	95% CI	Test proportion	Z-statistics	P (2-Sided)
Surgery	18	100%	81.47%–100.00%	0.5	4.243	<0.0001
No Surgery	0	0.00%				
Total	18	100%				
**Large Ear Keloids**						
	N	Proportions	95% CI	Test proportion	Z-statistics	P (2-Sided)
Surgery	131	72%	64.86%–78.41%	0.5	5.920	<0.0001
No Surgery	50	28%				
Total	181	100%				

## Discussion

Surgery is a commonly practiced therapeutic intervention for removal of ear keloids. Based on the findings of this study, the author proposes the following designations for keloid lesions.

### Primary ear keloids

A primary ear keloid is a keloid that has not been previously treated with surgery. Keloid lesions can form in any part of the ear; however, the location of the keloid solely depends on the site of the prior injury or ear piercing. All primary ear keloids start as a small skin lesion and grow over time. The longer a keloid is present, the larger it will become. Figure 5 depicts several examples of primary keloids in various stages of development.

### Secondary ear keloids

A secondary ear keloid is a new keloid that forms at the site of surgery for the removal of a primary keloid. Figure 2, Figure 3, and Figure 4 depict numerous cases of secondary ear keloids.

It is undisputable that the extent of the injury from the surgical removal of a primary ear keloid is significantly greater than the injury sustained from ear piercing. It is also logical to conclude that the extent of skin injury has a direct and linear relationship with the size and mass of keloid lesions. These two simple facts explain why keloid removal surgery can trigger development of larger keloids. Cognizant of the fact that there are patients whose keloids do not recur after surgery, we must be well aware, and acknowledge the deleterious effects of surgery, and the nightmare that is imposed on patients who end up developing large, semi-massive or massive ear keloids.

Tanaydin *et al.* reported their data on utilization of pressure devices ^6^ as adjuvant treatment after surgical excision of ear keloids, concluding that “keloid scars did not recur in 70.5% of treated patients”. This success rate, however, corresponds to a recurrence rate of 29.5%, almost one in three patients.

**Figure 8.**
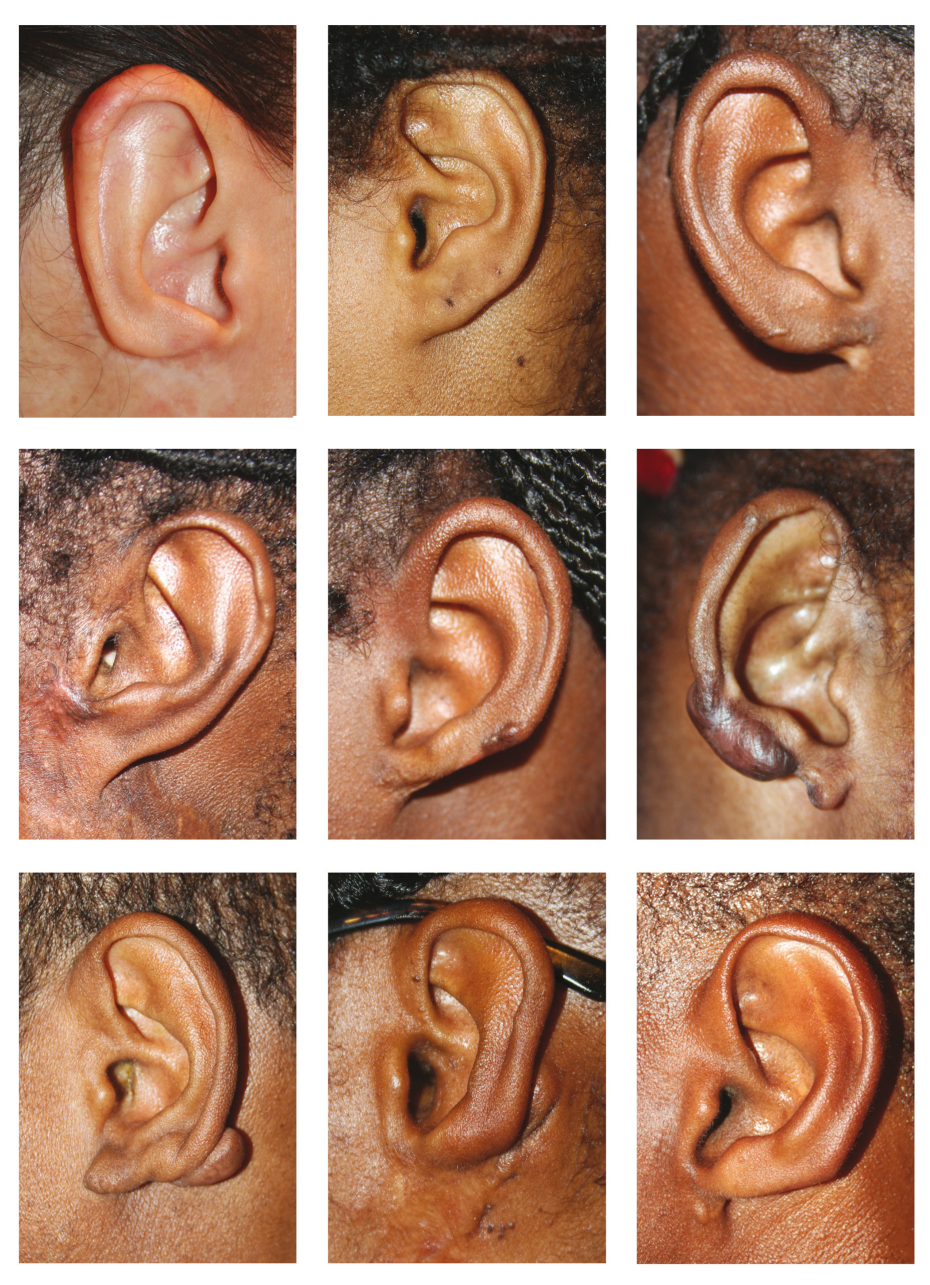
Poor surgical outcomes for which the true incidence remains unreported. Notice the disfiguration of normal ear anatomy and the loss of ear tissue from prior surgeries.

Recently-advocated approach of surgery in combination with adjuvant radiation therapy ^7, 9^, although it may yield to a lower keloid recurrence rate, it is by no means curative and still results in recurrence of keloids. It also exposes all patients to potentially grave adverse effects of radiation therapy.

Shin *et al.*, conducted a meta-analysis of the published data on adjuvant post-operative intra-lesional triamcinolone and radiation therapy after surgical excision of ear keloids and concluded that recurrence rates of 15.4% with adjuvant triamcinolone and 14.0% with adjuvant radiation therapy ^8^. These recurrence rates correspond to treatment failure in almost one in six or seven patients. Massive and semi-massive keloids occur in these exact subsets of patients who fail to respond to the best surgical efforts.

Indiscriminate and repeated surgical attempts to remove ear keloids are also associated with disfigurement of the ear. By attempting to remove the entire keloid, surgeons remove part of the earlobe or performs a wedge resection and remove some of the ear cartilage and soft tissue adjacent to the keloid. Even if this approach does not lead to the recurrence of the keloid, which it often does, it will result in the loss of normal ear anatomy and a poor aesthetic outcome. Figure 8 depicts several examples of such poor outcomes. A very common shortcoming of several publications on the surgical treatment of ear keloids ^2– 5^ is lack of reporting of the aesthetic outcomes.

The psychological stress and anxiety that is imposed on a young person by having to live with a worsened ear keloid is very real and life changing ^10^. Those of us who take on the task of treating keloid patients, often teenagers and young adults, need to be very cognizant about the risks associated with the treatments that we offer to our patients. Although surgery provides a quick-fix solution to an ear keloid, exposing children and young adults to a procedure that has even 1% risk of causing massive or semi-massive ear keloid is unacceptable, let alone a 11% risk that is observed among 283 consecutive cases presented here. It is unfortunate that the data on incidence of massive or semi-massive ear keloids has never been published, but a rate of 11% among author’s patients is very disturbing and resonates like a loud siren calling for more careful analysis of outcome data of all surgical interventions.

Furthermore, the carcinogenic risk of radiation therapy is real and should not be under-estimated. Let us not forget the fact that practice of radiation therapy for treatment of acne vulgaris was abandoned several decades ago, with some dermatologists referring to usage of radiation for treatment of benign skin conditions as “criminal” ^11^. Exposing teenagers and young adults to such a treatment, even with a small long term risk, is simply unacceptable. We need to bear in mind that we are not treating elderly cancer patients with radiation; we are treating teenagers and young adults. No matter how well the ear tissue is isolated and shielded, many thousands of hematopoietic stem cells that circulate in the capillaries and venules of the ear tissue will be exposed to ionizing irradiation. The author doubts that even one radiation therapist will be willing to expose his or her own ear tissue or that of his or her child to the radiation that is so casually offered to many young adults with KD.

Moreover, the real rate of keloid non-recurrence after adjuvant radiation therapy remains unknown. Most studies report their outcome after a short interval of few months to two years. A recent comprehensive review of adjuvant radiation therapy ^12^ for treatment of keloid lesions screened 207 publications, many of which were excluded for not describing a minimum length of follow up. The authors limited their study to 33 articles with only 10 studies providing incidence of recurrence. The mean time to recurrence was 14.8 ± 6.7 months with a range of 2–36 months post-treatment. True long term recurrence rate of keloids after adjuvant radiation therapy remains unknown. Author is currently treating a patient with massive left ear keloid who had her first recurrence 13 years after receiving adjuvant radiation therapy. Figure 2 depicts several cases of massive ear keloids in patients who had previously received adjuvant radiation therapy after surgical removal of their ear keloids.

## Need for a paradigm shift in treating primary ear keloids

**Figure 9.**
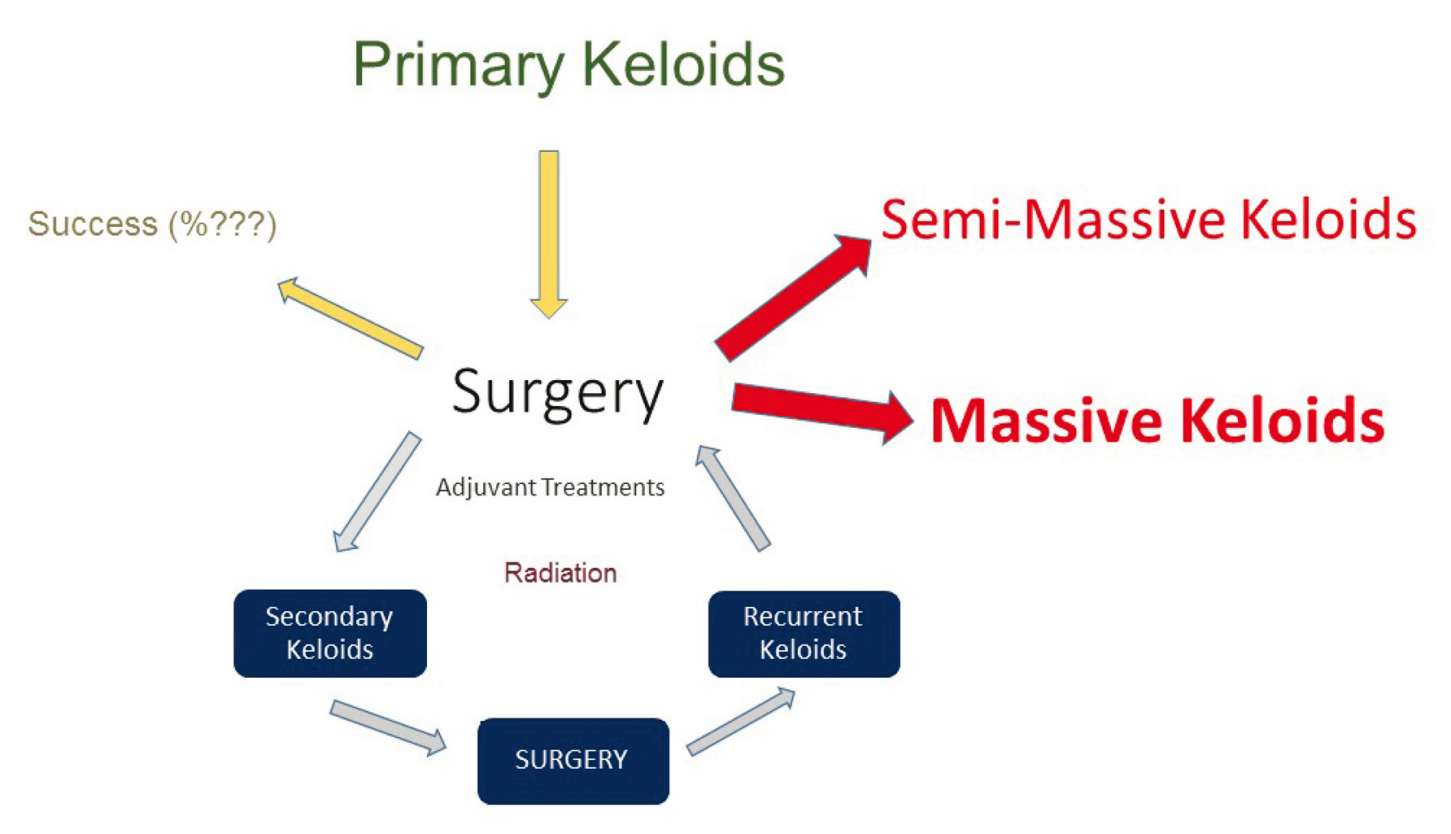
Vicious cycle of keloid removal surgery that results in the formation of semi-massive and massive in ear keloids.

The successful treatment of human diseases is reliant on thorough understanding of the underlying processes that lead to the development of particular illnesses. The basic principal of treating keloidal lesions is the destruction of the abnormal tissue with a method that will not trigger the underlying keloidal wound healing response. Surgical removal of keloids will indeed trigger this pathological wound healing response and can result in development of a much larger ear keloids. Figure 9 depicts the vicious cycle of surgery that can results in formation of semi-massive and massive ear keloids; a cycle that all 31 patients in this study, and all those shown in Figure 2 and Figure 3 have been through.

**Figure 10.**
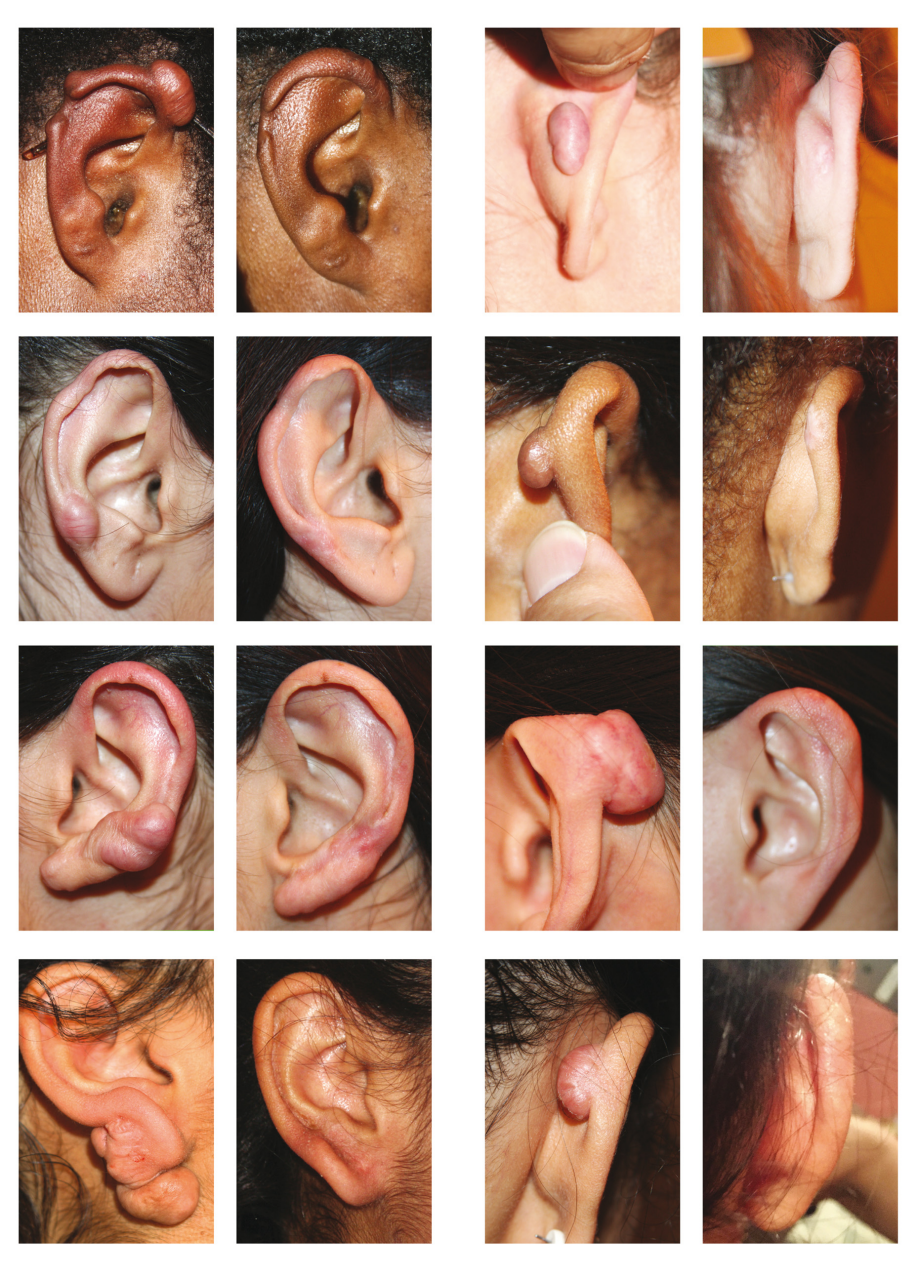
Successful keloid removal with contact cryotherapy for small primary and secondary (left column, bottom two cases) keloids. Notice the very minimal scarring at the site of cryotherapy. Most of these patients have enjoyed very durable and persistent results.

The development of all secondary keloids can be effectively prevented if we simply stop performing surgery on keloid patients all together. In the author’s opinion, supported by his own experience, the paradigm shifting treatment approach is a move to utilize contact cryotherapy for treatment of all primary ear keloids. Furthermore, the author believes that proper application of cryotherapy can effectively remove all primary ear keloids and prevent development of all secondary keloids. Results with high therapeutic success rate have been previously reported by others ^13– 16^. Figure 10 depicts several examples of durable results achieved by the author for patients with primary ear keloids.

Cryotherapy should be delivered properly and repeated as many times as needed. Liquid nitrogen is best applied to the keloid tissue using a properly sized hand-held applicator; such as a large cotton swab. The process should be repeated until the entire mass of keloid is frozen to the level of normal ear tissue. Within a few hours, the treated tissue becomes edematous and swollen and often forms a blister which frequently bursts and oozes serous fluid for several days. During this time frame, the treated keloid should be attended to as an open wound; thus, it is best covered with gauze and a loose dressing. Within the next several days, the treated tissue will become dehydrated and form a black-colored scab, which will remain in place for a few weeks. The scab will then gradually slough off. This process takes two to three weeks for very small keloids and up to six to eight weeks for larger keloids. In the author’s experience, upon recovery from the first treatment, most keloids show 30–60% reduction in their mass. Cryotherapy should be repeated, often every four to eight weeks in the same fashion until the keloid mass is totally destroyed. Depending on the size of the primary keloid, this process takes four to eight months and results in total removal of the primary keloids in almost every patient. Pressure devices or intra-lesional steroids should be used in all patients who continue to have a keloid remnant within their ear tissue.

Pain control is critical during the application of cryotherapy as well as during the first 24 hours after treatment. Inadequate pain control will result in a lack of compliance and poor treatment outcome. All patients should be educated about the process, and prescribed proper pain control medications.

Furthermore, there is no need to perforate the body of keloid with a very large bore metallic cannula, and to run liquid nitrogen through the core of keloid ^17^. There exist no data, and no evidence to support superiority of this invasive technique to standard, non-invasive contact cryotherapy.

Although we can successfully debulk and remove almost all primary ear keloids with cryotherapy, there is a clear need for follow up in all patients in order to detect and treat early recurrences. Repeat cryotherapy, intra-lesional steroid and/or intra-lesional chemotherapy should be considered in treating keloid recurrence.

Performing surgery to remove primary ear keloids is inherently contrary to both of the above principles. Surgery, by its nature, induces new injury to the skin, and as shown in Figure 8, the surgical removal of a primary keloid frequently results in the loss of surrounding normal ear tissue. The loss of normal ear tissue, even in the absence of future keloid recurrence, will often result in an unacceptable aesthetic outcome. The worsening of ear keloids after surgical excision is caused by the triggering of the same dysregulated wound healing response, yet to a new dermal injury that is more extensive in nature than the injury from the ear piercing itself.

There are many circumstances – but most importantly when surgery is performed on a patient – that we have the duty to obtain “informed consent” and not just a “permission to operate”. Simple disclosure of the general risks associated with a surgical procedure is clearly inadequate. It is our professional duty to advise all keloid patients of the specific risks that are associated with the surgical removal of their keloids. We are obligated to disclose the risk of developing massive and semi-massive keloids to each and every patient who is advised to undergo surgery. Through the informed consent process, we have the ethical and moral obligation of showing the images of massive and semi-massive ear keloids to our patients, and informing them that with keloid surgery, there is a risk of developing such life-changing complications. We are also obligated to discuss alternative procedures, or conservative nonsurgical approaches. As healers who are licensed to practice medicine, we have the obligation of respecting clinical data, disease biology and the process of informed consent, all of which translate into improvement in: treatment outcomes for our patients.

## Conclusions

Although this study is limited by its size, and patients were drawn from only one medical practice that does not offer surgery for treatment of keloids, several interesting factors stand out as risk factors for development of large, semi-massive and massive ear keloids.

□ Prior keloid removal surgery was the most important risk factor for development of massive and semi-massive ear keloids. Without exception, all these patients had undergone anywhere between one to seven prior keloid removal surgeries.
□ Prior keloid removal surgery was the most important risk factor for development of large ear keloids with 72% of patients having prior keloid removal surgery.
□ African/African American race was noted to be a major potential risk factor in all four groups, most importantly among those with massive, semi-massive ear keloids, with only five Caucasians/Asians among the 31 patients (11%) in both these groups.

## Recommendations

The goal of treatment for keloid lesions, and ear keloids in particular, should not only focus on removal of the keloid tissue, but most importantly on two other very important principles:

1. Prevention of damage to the ear tissue
2. Prevention of the recurrence of the keloid

Topical contact cryotherapy should be the primary mode of treatment for all primary and secondary ear keloids. This approach will prevent the development of incurable secondary and, large, semi-massive and massive keloids and eliminate the need for hazardous adjuvant radiation therapy.

## Data availability

The data referenced by this article are under copyright with the following copyright statement: Copyright: © 2017 Tirgan M

Data associated with the article are available under the terms of the Creative Commons Zero "No rights reserved" data waiver (CC0 1.0 Public domain dedication). 

All raw data relevant to the study are provided in tables above.
