# Supplementary material for: Massive ear keloids: Natural history, evaluation of risk factors and recommendation for preventive measures – A retrospective case series
Source: F1000Res. 2017 Mar 21;5:2517. Originally published 2016 Oct 13. [Version 2] doi: 10.12688/f1000research.9504.2 (PMC5428534; doi:10.12688/f1000research.9504.2)
Supplement: Peer review history

# Peer review: Referee response for version 2

**DOI:** 10.5256/f1000research.11970.r22098

**Published:** 2017-05-05

**Authors:** Mohamed Badawy Hassan Tawfik Abdel-Naser

**Affiliations:** 1 Department of Dermatology, Andrology and STIs, Faculty of Medicine, Ain Shams University, Cairo, Egypt

This is a retrospective study in which the author examined the demography of keloid patients. The following points need consideration.

1. The manuscript is too long and it can be markedly reduced without any impact on the quality of the study.  For example
  a. Fig. 1. No need for this figure in the present study and it can be omitted together with its relevant text. Both can be useful for a separate review article.
  b. In the results section, patients data are given in the text and repeated in table. 1. This is unnecessary repetition. In general, what is mentioned in text should not be mentioned in table and vice versa. In fact, author can insert all patients' data in a single table.   
  c. Table 2. Author gave a short account of another staging system of keloid which i believe more reliable (as it is objective) than the subjective visual method of the present study. However, what is the rational of mentioning this method and table 2 in this study? Both can be omitted.
  d. Tables 3 and 4 can be easily incorporated in a single table without many details, such as p values. Significance can be marked with asterisk (e.g., for gender), dagger (e.g. for race), etc as a footnote. In fact both can be incorporated in table 1.
  e. Figures 6 and 7 can be combined in one figure and the significant columns labelled (with asterisk, dagger etc) and amended as a footnote. 
2. The aim of the study is not clear and perhaps should be added in a clear statement (s).
3. The author suggested that age of patients and duration of keloid are important factors, but the study lacks any correlation with these 2 parameters.
4. The discussion section is not focusing on the results of this retrospective study. Is there any reason (s) apart from genetics to explain the significant tendency of Africans to develop keloids? Are fibroblasts of Africans different from Caucasians? Is there any additional explanation(s), apart from ear piercing, for the gender difference in keloid formation? The author is not in favor of surgical removal of keloids because of the risk of recurrence and massive keloid formation. Is this risk solely related to surgical injury? Does the skill of the surgeon play a role?  Does the suture material or postoperative wound infection contribute to this unfavorable outcome? In general there are several confounding factors that need to be discussed instead of elaborating on other methods not related to this retrospective study.  
5. The mentioned recommendations are not appropriate in this study. Author can work on separate review article to discuss in details the different therapeutic modalities of keloids and the pros and cons of each method. In the retrospective study, the author is expected to discuss his findings.

I have read this submission. I believe that I have an appropriate level of expertise to confirm that it is of an acceptable scientific standard, however I have significant reservations, as outlined above.

# Peer review: Referee response for version 2

**DOI:** 10.5256/f1000research.11970.r21592

**Published:** 2017-04-06

**Authors:** Robert Sidbury

**Affiliations:** 1 Seattle Children's Hospital, University of Washington School of Medicine , Seattle, WA, USA

I appreciate Dr Tirgan’s replies. I certainly endorse his overarching messages that injury to the skin is a major predisposing factor to keloid development, and surgery is certainly a form of injury to the skin. I also wholeheartedly endorse the need for a full discussion with affected patients about the full range of treatment options, both medical and surgical, and the risks and benefits of both. I continue to feel that while cryotherapy is indeed an option, and clearly a successful one in many cases, it is not viable for all and in some cases surgery can be the best choice with the aforementioned caveats. As for the lack of a billing code for cryotherapy as a possible reason for this not being offered to patients, I guess I can’t speak for all who practice in this realm but only myself: I don’t ever personally operate on keloids nor have any financial connection to those who do so my own motivation is not so conflicted. I can and do bill for cryotherapy, keloids, warts or other, when appropriate. All of that said, this is a very nice review of the topic and will no doubt be useful to providers (and patients) as they consider their options.

I have read this submission. I believe that I have an appropriate level of expertise to confirm that it is of an acceptable scientific standard, however I have significant reservations, as outlined above.

# Peer review: Referee response for version 2

**DOI:** 10.5256/f1000research.11970.r21414

**Published:** 2017-03-31

**Authors:** Amy J. McMichael

**Affiliations:** 1 Department of Dermatology, Wake Forest School of Medicine, Winston-Salem, NC, USA

I reviewed the revision of the Tirgan article and it appears much more clearly organized. Results are in the results section. The discussion points are based upon actual data. The manuscript is quite long, but the slides are great.

I have read this submission. I believe that I have an appropriate level of expertise to confirm that it is of an acceptable scientific standard.

# Peer review: Referee response for version 1

**DOI:** 10.5256/f1000research.10237.r19062

**Published:** 2017-01-09

**Authors:** Amy J. McMichael

**Affiliations:** 1 Department of Dermatology, Wake Forest School of Medicine, Winston-Salem, NC, USA

This is a good accounting of keloids with many cases for discussion. The placement of new data in the discussion section is a bit off-putting for the reader. I would recommend placing all the charts in the Results section rather than having them in the Methods Section. Also, there needs to be more than just the grouping of keloids as presented. Basically, this is a list of keloids. There needs to be some correlation drawn or statistics to note associations rather than just descriptive stats. This is a great start, but just needs more to really give the reader information that is useful. I recommend not putting new data in the discussion and conclusion and moving this to the results.

 The analysis used on the cases should be included in the methods section. The discussion should focus on the natural history of all the things that are discussed in the results sections.

I have read this submission. I believe that I have an appropriate level of expertise to confirm that it is of an acceptable scientific standard, however I have significant reservations, as outlined above.

# Peer review: Referee response for version 1

**DOI:** 10.5256/f1000research.10237.r17447

**Published:** 2016-11-15

**Authors:** Robert Sidbury

**Affiliations:** 1 Seattle Children's Hospital, University of Washington School of Medicine , Seattle, WA, USA

Overall this is a nice review.  There are certain small additions I would make (e.g. a line somewhere capturing the idea that keloids can be seen in certain syndromes disproportionately such as Rubinstein-Taybi syndrome). This might reasonably be placed in the abstract (e.g. KD is an inherited wound healing ailment, frequently seen among Africans/African Americans, Asians, and in some genetic disorders such as Rubinstein Taybi syndrome). A notation like this in the introduction would also make more complete. 

 The aforementioned criticism pales in comparison with my overarching concern that this article has more of an agenda than just information dissemination. The agenda is that surgery is bad for ear keloids and the author does not shy away from this opinion. My concern is not that he expresses this opinion, even advocates for it to a certain extent if he believes it to be true, but the lengths he takes this, and the way he uses his "data" asymmetrically to make this point gives me pause. 

 First, what about the point itself? Is it valid? Is surgery bad for keloids? Yes, surgery is an injury to the skin which can itself promote keloid formation, indeed so much so that our surgeons will not operate to remove or debulk keloids in certain areas (eg neck, trunk). However, they do believe and I have shared plenty of patients to validate that ear keloids can be effectively removed in a sustainable, cosmetically acceptable way without necessarily (and in fact rarely) resorting to radiation therapy adjunctively. This sort of response is well-documented in the literature they just don't happen to be references this author cites:

- Triamcinolone after surgery as effective as radiation therapy (and both can be effective) at preventing recurrence ^1^
- Pressure clips after surgery can prevent recurrence (this is technique used by my colleagues in Plastic Surgery to good effect) ^2^

There are countless other references of success using surgery with acceptable risk-benefit profiles.

 Second, where do I believe the author goes beyond simply stating his opinion, backed up by his data, that surgery can lead to recurrence and poor outcome? 

- First paragraph page 3, final sentence, "...unfortunately, ear keloids will continue...". This is stated as if it is ALWAYS the case and it simply is not. 
- Why did he not include post-otoplasty patients in study?
- p 8 Results: Don't have experience with their own surgical successes because none of these patients have that modality ("do not offer surgery for treatment of keloids"). Therefore the only patients they see are surgical failures.
- p 8 under Secondary Ear Keloids: "Cognizant of the fact that there are patients whose keloids do not recur after surgery..." and then there is no balance in what follows. This context that author describes speaks to the need for an appropriate and careful risk-benefit discussion but not necessarily not offering surgery at all when there are many reasons why it is sometimes the right choice.  What if the keloid itself is life altering? What if they do not have time to return for repeated liquid nitrogen over the course of a year? With the attendant blistering and healing phases played out with each treatment? And the cost? 
- p 10 paragraph 2: "1% risk of causing massive or semi-massive ear keloids is unacceptable" all depends on patient and context.
- Figure 6: "after" shots should be paired with "before" shots; I can imagine at least some of these outcomes potentially preferable to keloid it replaced but can't know without seeing images.
- p 10: Need for a paradigm paragraph # 1. Stop all surgery, cryo for all...this just isn't practical or medically justifiable.
- p 10 cryotherapy paragraph: This just isn't possible for all patients. Problems include time, $, quality of life during treatment, incomplete response because not all patients respond to cryo; post inflammatory pigment alteration secondary to cryo especially in darker skinned patients which this author does not mention at all.

So, in summary, this is simply too one-sided and agenda driven to be an appropriate publication in my opinion. Could it be modified? Sure. Present cryotherapy and other options alongside surgery and feel free to opine but a more balanced presentation with updated references would be required for me to endorse.

I have read this submission. I believe that I have an appropriate level of expertise to confirm that it is of an acceptable scientific standard, however I have significant reservations, as outlined above.

## References

[1] ShinJYLeeJWRohSGLeeNHYangKM: A Comparison of the Effectiveness of Triamcinolone and Radiation Therapy for Ear Keloids after Surgical Excision: A Systematic Review and Meta-Analysis. *Plast Reconstr Surg*.2016;137(6) : 10.1097/PRS.0000000000002165 1718-25 10.1097/PRS.0000000000002165 27219228

[2] TanaydinVBeugelsJPiatkowskiACollaCvan den KerckhoveEHugenholtzGCvan der HulstRR: Efficacy of custom-made pressure clips for ear keloid treatment after surgical excision. *J Plast Reconstr Aesthet Surg*.2016;69(1) : 10.1016/j.bjps.2015.09.013 115-21 10.1016/j.bjps.2015.09.013 26507863

# Response

**Published:** 2016-11-18

**Authors:** Michael Tirgan

**Affiliations:** Keloid Research Foundation, USA

Dear Dr. Sidsbury:

 Thank you very much for taking the time to review and comment on my publication. Peer review plays an important role in publishing research material. I truly appreciate your detailed and thorough review and each and every comment you have made.   I hereby address the points you have raised.

1. As for referencing to Rubinstein-Taybi Syndrome - This manuscript is focused on providing data about Massive Ear Keloids. It is by no means review of the disorder. 
2. As for your comment about my point being that “surgery is bad for ear keloids” – I am personally convinced that to be the case. Keloid is a genetic disorder that involves much of the normal appearing skin. It is not limited to the area of a keloid growth, therefore surgery cannot cure it. There are numerous publications that have eloquently explained this fact. This conclusion is backed by data as presented in the manuscript.  It is also backed by clinical observation of some physicians, and parents and relatives of keloid patients, who dissuade patients from undergoing surgery. 
3. As for your comment “therefore the only patients they see are surgical failures” - this is simply not correct. Patients shown in Figure 5 are some of my patients who presented with very early stages of ear keloid and had chosen a non-surgical approach. As for breakdown of the study cohorts, there were 283 patients in the study. 31 patients had massive or semi-massive ear keloids. Among 181 patients with large keloids, only 73% had prior surgery and 27% rest of these patients never had surgery. Of the 71 patients in the “small ear keloid” majority had not undergone surgery. Altogether, more than a third of all patients did not have surgery. 
4. As for your comment about this manuscript being “agenda driven” - I simply disagree with you. My conclusions are rather data driven. We - as physicians and as healers – have the ethical and moral obligation of providing our patients with the best available treatment. The Declaration of Geneva of the World Medical Association binds us with the words, “The health of my patient will be my first consideration,” and the International Code of Medical Ethics declares that, “A physician shall act in the patient's best interest when providing medical care.”. It is the duty of the physician to promote and safeguard the health, well-being and rights of patients.  
  The Declaration of Geneva also states that “The primary purpose of medical research involving human subjects is to understand the causes, development and effects of diseases and improve preventive, diagnostic and therapeutic interventions (methods, procedures and treatments). Even the best proven interventions must be evaluated continually through research for their safety, effectiveness, efficiency and accessibility." Purpose of this study was to exactly do what the Declaration of Geneva was intended us to do. Data provided in the manuscript points to the shortcomings of surgery by way of causing massive and semi-massive keloids. Manuscript provides argument as to why this may be the case. All conclusions are driven by data.
5. As for your comments about surgery that “This sort of response is well-documented in the literature they just don't happen to be references this author cites”, I would refer to the two references provided at the conclusion of your comments. 
  Shin et. al reported “The recurrence rate after surgical excision of an ear keloid in the triamcinolone group was estimated as 15.4 percent. The recurrence rate in the radiation therapy group was estimated as 14.0 percent.”
  Tanaydin et. al reported “Keloid scars did not recur in 70.5% of treated patients”. By doing the math, the recurrence rate was 29.5% among patients who used custom made pressure clips after surgery. 
  There is no doubt that some patients develop massive and semi-massive keloids - the ones who fail to respond to the best surgical efforts - i.e. the 14-15% (one in six patients) reported by Shin and 29.5% (one in three) reported by Tanaydin.
  I hope to see more publications about the incidence of massive and semi-massive ear keloids. As of this date, searching pubmed does not locate even one publication about incidence of massive ear keloids.
  I do advocate cryotherapy as primary treatment of all bulky ear keloids. There are several references that lend support to the usage of cryotherapy for treatment of ear keloids. Cryotherapy, as a treatment modality for keloids is also mentioned in every textbook of dermatology and in every overview of keloids, however, hardly any dermatologist or plastic surgeon uses it.I wonder why?I know for fact that there are no billing codes for application of cryotherapy for treating keloids.
6. As for “not including post-otoplasty cases” – there were only 14 patients. I have written is separate manuscript about this cohort. 
7. As for figure 6 – the point was to show less than ideal aesthetic outcome of ear keloid surgery.
8. As for paradigm shift to stop surgery – I think it can be done. Although surgery is a quick-fix and results in immediate removal of ear keloids, it does cause long term harm to many patients. To this date, we do not have a methodology to identify who will be the next patient at risk of developing a massive ear keloid after surgical removal of a small primary ear keloid. In my data set, the rate is close to 11%. To know the exact risks and outcomes, we need establish a Keloid Surgery Registry, and register each and every keloid patient who undergoes surgery, and follow them for several years. In absence of such data, or data from a well-designed randomized trial, all we can do is to inform our patients of the potential risks of keloid surgery.
  There are many circumstances – but most importantly when surgery is performed on a patient – that we have the duty to obtain “informed consent” and not just a “permission to operate”. We also have the duty to advise our patients of the specific risks of the procedure. We do know that disclosure of the general risks that are associated with any surgical procedure is not adequate. We are obligated to disclose the risk of developing massive and semi-massive keloids to each and every patient who is to undergo keloid removal surgery. We are also obligated to discuss alternative procedures, or conservative nonsurgical approaches with our patients. Through informed consent process, we have the ethical and moral obligation of showing the images of massive and semi-massive ear keloid to their patients, and informing them that with keloid surgery, there is a risk of developing such life-changing complications.
9. As for the cost, risks, rate of skin discoloration and other aspects of cryotherapy, only a well-designed randomized study to compare surgery to contact cryotherapy will be able to answer all these valid points. I hope that there will be enough interest to support and fund such a study. 
10. Finally, I am of the view that we all should respect data, disease biology and the process of informed consent. I only wish to improve the outcome of our patients. I hope that through collaborations with physicians like yourself, we can join forces and tackle this very hard-to-treat disorder.

Thank you again for all your comments.

 Michael H. Tirgan, MD
